# Oxidative regulation of the mechanical strength of a C–S bond†

**DOI:** 10.1039/d0sc04381h

**Published:** 2020-09-14

**Authors:** Yangju Lin, Stephen L. Craig

**Affiliations:** Department of Chemistry, Duke University Durham North Carolina 27708 USA stephen.craig@duke.edu

## Abstract

The mechanical strength of individual polymer chains is believed to underlie a number of performance metrics in bulk materials, including adhesion and fracture toughness. Methods by which the intrinsic molecular strength of the constituents of a given polymeric material might be switched are therefore potentially useful both for applications in which triggered property changes are desirable, and as tests of molecular theories for bulk behaviors. Here we report that the sequential oxidation of sulfide containing polyesters (**PE-S**) to the corresponding sulfoxide (**PE-SO**) and then sulfone (**PE-SO2**) first weakens (sulfoxide), and then enhances (sulfone), the effective mechanical integrity of the polymer backbone; **PE-S** ∼ **PE-SO2** > **PE-SO**. The relative mechanical strength as a function of oxidation state is revealed through the use of *gem*-dichlorocyclopropane nonscissile mechanophores as an internal standard, and the observed order agrees well with the reported bond dissociation energies of C–S bonds in each species and with the results of CoGEF modeling.

## Abstract 2

The mechanical strength of individual polymer chains is believed to underlie a number of performance metrics in bulk materials, including adhesion and fracture toughness.

## Graphical abstract

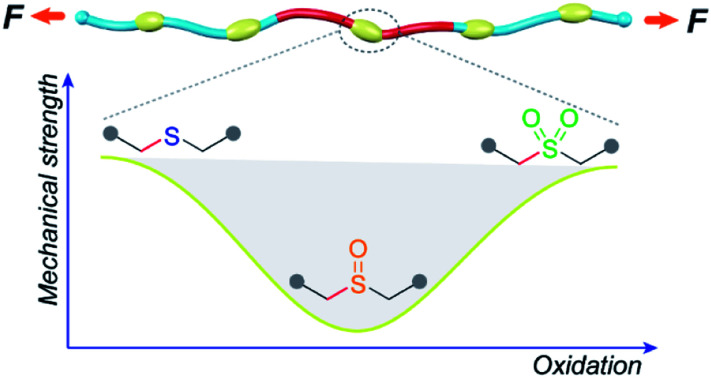

## Introduction

The mechanochemical scission of individual polymer chains limits their individual toughness, and it is also hypothesized to directly impact macroscopic material properties including, in some cases, the critical performance metrics of fracture toughness and adhesion.^1^ Strategies for the *in situ* switching of the intrinsic molecular strength of a given polymeric material are therefore attractive on two fronts: (i) as a mechanism through which stimuli-responsive mechanical properties might be achieved; and, (ii) as a direct probe to test long-held molecular theories for bulk behaviors (*e.g.*, the Lake–Thomas theory^2^). To date, the external regulation of mechanochemical scission has been achieved using a photo-adaptable diarylethene-conjugated Diels–Alder adduct.^3^ Inspired by this report, we sought an externally switchable mechanophore, with the following design parameters in mind: (i) minimal size; (ii) ease of synthesis; (iii) preceding use in bulk materials synthesis; (iv) responsiveness to stimuli other than light, in order to complement the prior work.

To this end, we hypothesized that controlling the oxidation of sulfide, which can be conveniently incorporated into polymers, provides an opportunity to achieve oxidative regulation of the mechanical strength of a C–S bond. Relative to polymeric systems that respond to light,^4^ heat,^5^ pH,^6^ force,^7^ and other stimuli,^8–10^ polymers whose properties are responsive to oxidation state play remarkable roles in controlled assembly,^11^ self-healing ability,^12^ adjusting gel volume,^13^ and drug delivery.^14–16^ Construction of such polymers is achieved through incorporation of oxidizable or reduceable (including, in some circumstances, those can be reversibly switched between two oxidation states) chemical functional units, including ferrocene,^17^ selenide/diselenide,^18,19^ platinum complexes, sulfide, aryl oxalate esters, phenylboronic esters, thioketals, proline, *etc.*^15^ Among these systems, we were drawn to sulfide containing polymers, which are easily constructed through thiol–ene “click” reactions,^20^ Michael additions,^21^ ring-opening of ethylene/propylene sulfide^22,23^ and other scalable and accessible chemistry pathways.^24,25^ Sulfide-based polymers are further useful in fabricating materials that are capable of adapting nanomorphology,^26^ changing solubility,^27–29^ tuning mechanical modulus^30^ and conductivity,^31^ and delivering drugs when exposed to oxidants. Generally, the response is triggered by oxidizing sulfide to sulfoxide or sulfone, which is accompanied by a change in dipole moment/hydrophilicity.^32^

**Fig. 1:**
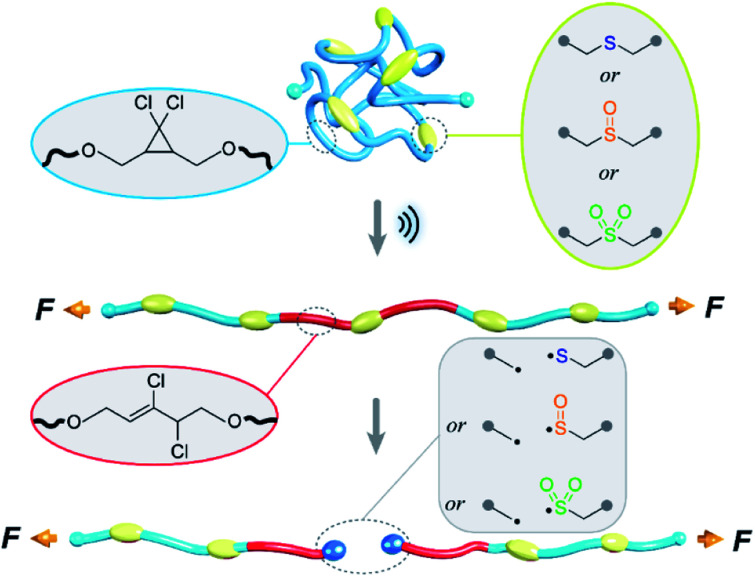
Illustration of competition between *g*DCC ring opening and C–S bond scission on polymer backbone under sonication. Relative mechanical strength of C–S bonds in sulfide, sulfoxide and sulfone were compared.

The oxidation to sulfoxide or sulfone affects the C–S bond dissociation energy,^33^ and we hypothesized that the corresponding mechanical strength (force required for the rapid mechanochemical scission of the corresponding C–S bond) might change similarly. The relative mechanical strengths of sulfide, sulfoxide, and sulfone can be quantified through the use of a nonscissile *gem*-dichlorocyclopropane (*g*DCC) mechanophore as an internal standard in pulsed sonication experiments.^34^ Relative to the conventional single chain-centered mechanophore strategy, for which the main challenge lies in the quantification of mechanophore activation,^35^ the use of multiple scissile mechanophores and nonscissile internal standards within the same polymer^36^ enhances the ability to detect differences in reactivity that might not be obvious from, for example, relative rates of chain scission in single-mechanophore polymers.^37^ As illustrated in Fig. 1, there exists a competition between *g*DCC ring-opening and C–S bond scission, and the extent to which *g*DCC ring-opening could occur before chain scission depends on the mechanical strength of C–S bonds. The percentage of *g*DCC ring opening (*g*DCC RO%) per chain scission cycle (SC, where SC = ln(*M*^(0)^_n_/*M*^(t)^_n_)/ln 2) is characterized by *Φ*, the slope of *g*DCC RO% *vs.* SC, and it indicates the relative mechanical strength of a *g*DCC containing polymer, *i.e.*, a lower *Φ* value means a weaker polymer chain. Mechanistic studies have revealed that the use of *Φ* value accounts for variations in temperature, solvent, concentration, sonication amplitude, *etc.*, but that the initial contour length (*M*_n_) of the polymer does matter, with shorter polymers having higher *Φ* values (*e.g.*, for a polybutadiene based *g*DCC polymer, *Φ* = 0.92 for *M*_n_ = 59 kDa *vs. Φ* = 0.69 for *M*_n_ = 92 kDa).^35^ This quantification strategy has been applied to studies of the relationship between covalent bond strength and mechanical strength,^38,39^ the mechanochemistry of metallocenes^40,41^ the chain dynamics of cyclic polymers^42^ under high strain rate elongational flows, and the subtle influence of stereochemical effects on the mechanical reactivity of Diels–Alder adducts.^37^

## Results and discussion

**Scheme 1:**
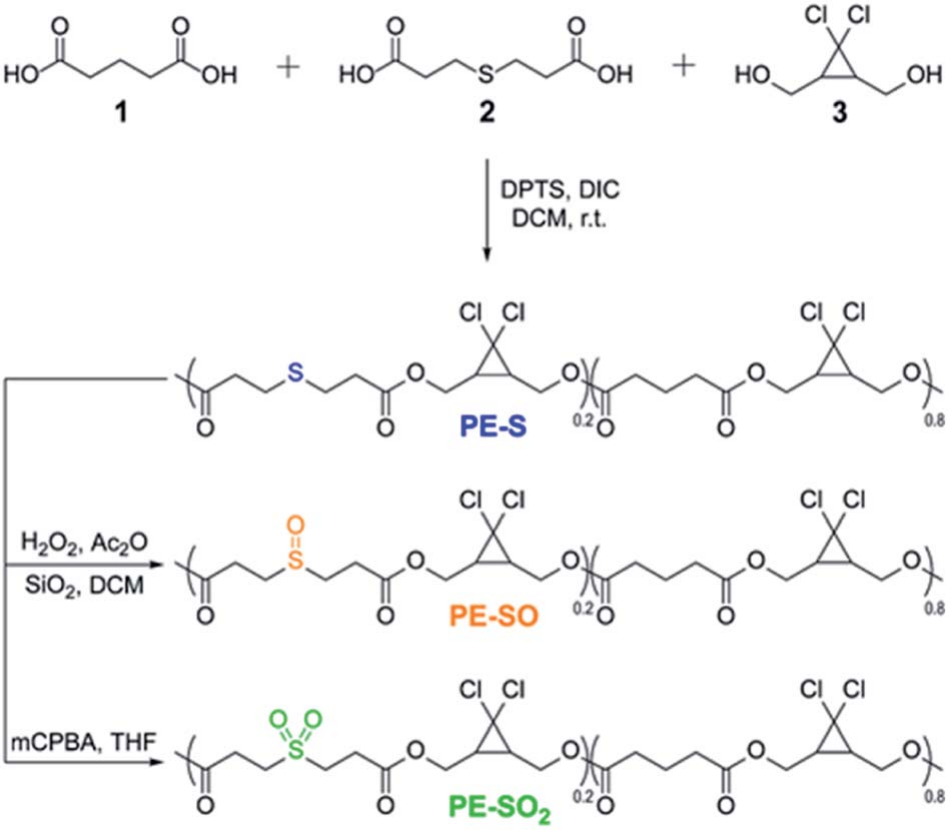
Synthesis of sulfide containing polymer and corresponding oxidation of sulfide to sulfoxide and sulfone.

We prepared multi-mechanophore *g*DCC and sulfide containing copolymers using a polyesterification strategy (Scheme 1).^37,43^ Copolymerizing glutaric acid **1**, 3,3′-thiodipropionic acid **2**, and *g*DCC diol **3** (molar ratio, **1**/**2/3** = 4 : 1 : 5) monomers gave a **PE-S** polymer containing the expected 20 mol% of sulfide repeats along the backbone. The greater the concentration of scissile mechanophore, the smaller the contribution from non-specific chain scission, and 20 mol% is typically more than sufficient to allow mechanical strength to be characterized as a function of bond strength^34^ or reaction stereochemistry.^37^**PE-S** was further oxidized to either the corresponding polysulfoxide (**PE-SO**) using a mild oxidation protocol^44^ or polysulfone (**PE-SO2**) using *meta*-chloroperoxybenzoic acid (*m*CPBA) as an oxidant.

**Fig. 2:**
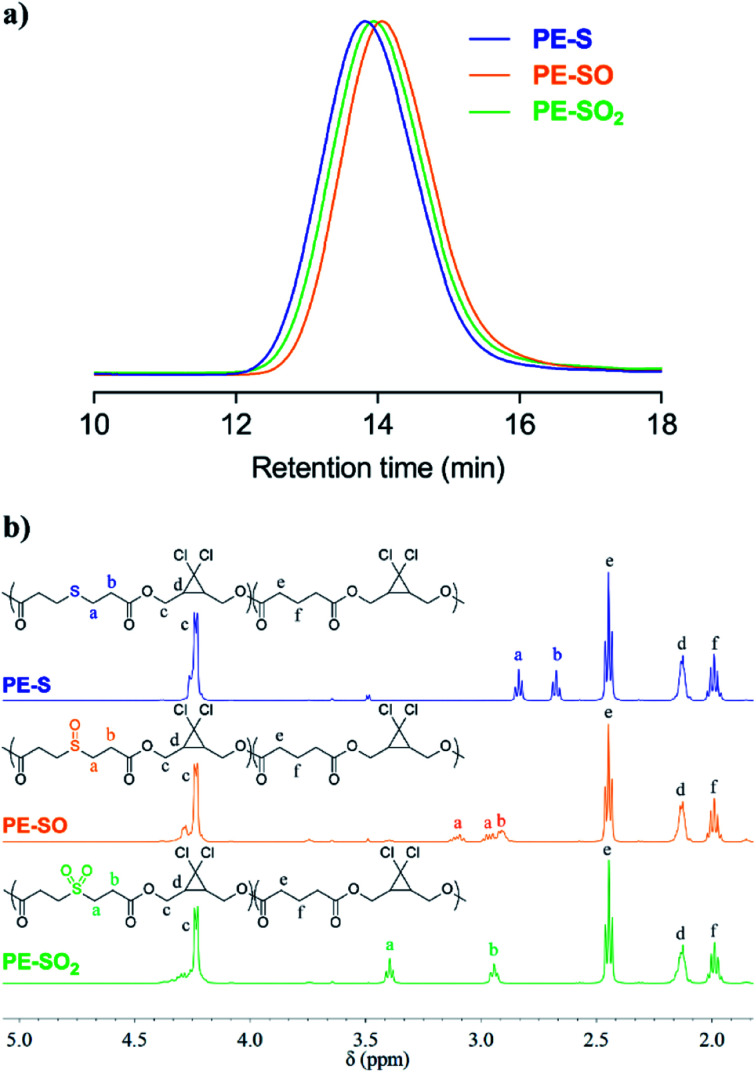
(a) Normalized GPC traces (RI signal, THF eluent) of **PE-S**, **PE-SO** and **PE-SO2**. (b) ^1^H NMR (500 MHz, CDCl_3_) spectra of **PE-S**, **PE-SO**, and **PE-SO2**.

Obtained polymers were analyzed by GPC (THF mobile phase) coupled with refractive index (RI) and multi-angle light scattering (MALS) detectors. As shown in Fig. 2a, oxidation of **PE-S** to **PE-SO** and **PE-SO2** leads to a shift in retention time from 13.81 min to 14.06 min, and 13.94 min, respectively. Molecular weights (*M*_n_) determined by MALS are consistent with the shifts in retention time; *M*_n_ = 72 kDa for **PE-S** (*Đ*_M_ = 1.45), *M*_n_ = 54 kDa for **PE-SO** (*Đ*_M_ = 1.48), and *M*_n_ = 60 kDa for **PE-SO2** (*Đ*_M_ = 1.48). We attribute the reduction in *M*_n_ on going from **PE-S** to **PE-SO** to low levels of oxidative degradation, and the increase in *M*_n_ on subsequent oxidation to **PE-SO2** to the addition of O atoms to the polymer chains.

The conversion of sulfide to sulfoxide and sulfone is verified by ^1^H NMR spectroscopy. The protons α and β to the sulfur atom (*H*_a_ and *H*_b_, Fig. 2b) begin as clean triplets at 2.84 and 2.67 ppm in **PE-S**. Upon oxidation, *H*_a_ evolves into two coupled multiple peaks at 3.10 and 2.96 ppm, and *H*_b_ shifts to 2.91 ppm, which agree well with reported values.^45,46^ The splitting of *H*_a_ is a result of the asymmetry of the sulfoxide, which renders the two *H*_a_ protons diastereotopic and magnetically inequivalent. Further oxidation to the symmetric sulfone restores the two triplet peaks, albeit at positions that are further downfield (*d* = 3.40 ppm and 2.94 ppm).^47^ Other peaks in the spectra remain effectively unchanged. Integration of *H*_a_/*H*_b_ relative to other backbone protons reveals that the molar content of sulfide, sulfoxide, and sulfone stays at a constant value of ∼20 ± 1 mol% (see ESI†), establishing the selective oxidation of sulfide to sulfoxide and sulfone.

**Fig. 3:**
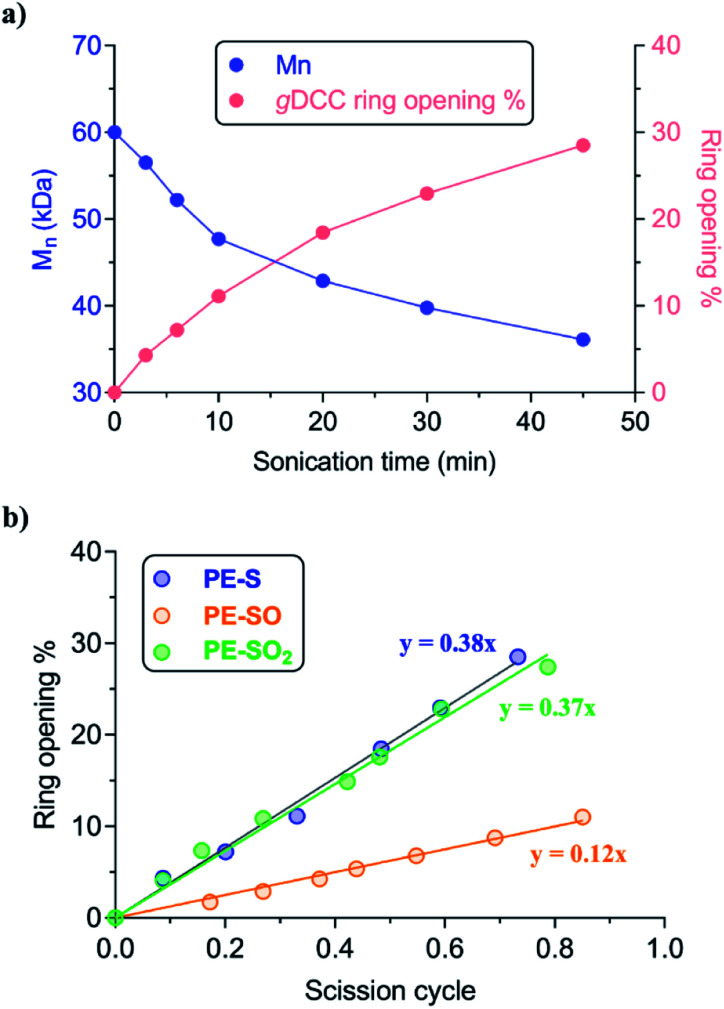
(a) The evolution of *M*_n_ in **PE-S** and corresponding percentage of *g*DCC ring opening at various sonication times. (b) The fraction of *g*DCC ring opening *vs.* scission cycle for each C–S containing polymer. **PE-S** (*M*_n_ = 60 kDa), **PE-SO** (*M*_n_ = 54 kDa) **PE-SO2** (*M*_n_ = 60 kDa).

Ultrasonication was used to quantify the relative mechanical strength of the polymer as a function of its oxidation state. The *M*_n_ of initial **PE-S** is 72 kDa, which corresponds to a greater contour length than is found in the direct products of its sequential oxidation: **PE-SO** (*M*_n_ = 54 kDa) and **PE-SO2** (*M*_n_ = 60 kDa). The initial *M*_n_ affects the *Φ* value and therefore complicates a direct comparison of C–S bond strength as a function of oxidation state in cases where a slightly lower *Φ* value is obtained for a longer polymer. Such is the case when comparing **PE-S** to **PE-SO2** (see above discussion and Fig. S5†), and so we investigated another **PE-S** polymer with *M*_n_ = 60 kDa (*Đ*_M_ = 1.44) to facilitate the comparison with **PE-SO** (*M*_n_ = 54 kDa) and especially **PE-SO2** (*M*_n_ = 60 kDa). Results obtained with the 72 kDa parent polymer, however, are consistent with the 60 kDa polymer and show that the variation in mechanical reactivity can be obtained through sequential oxidation within a given polymer. In a typical experiment, a THF solution of the polymer (2 mg mL^−1^) was treated with pulsed ultrasonication (30% amplitude, 1s on, 1s off, ice bath, N_2_), with aliquots removed and analyzed periodically until the *M*_n_ was reduced to nearly half of its initial value. As the *M*_n_ decreased, the extent of *g*DCC ring opening increased. For example, after subjection of **PE-S** polymer to ultrasonication for 45 min, the *M*_n_ drops from 60 kDa to 36 kDa, and this is accompanied by 29% *g*DCC ring opening along the polymer backbone (Fig. 3a). Here, polymer chain scission is ascribed to the selective C–S bond cleavage, based on previous evidence that the C–S bond is, mechanically, a relatively weak bond compared with other C–C and C–O bonds along the polymer backbone (also see discussion below).^34^ The chain scission cycle (SC) is calculated according to the following equation:
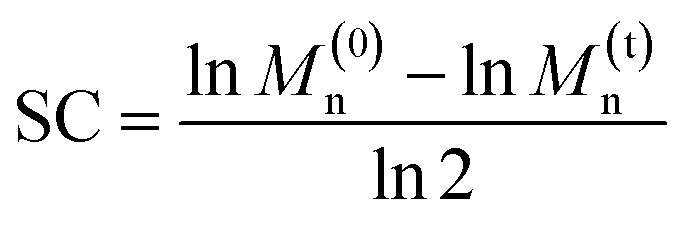
where *M*^(0)^_n_ and *M*^(t)^_n_ are initial and sonicated molecular weight, respectively.

The evolution of *g*DCC RO% *vs.* SC is shown in Fig. 3b. The *Φ* value of **PE-SO** is 0.12, *vs.* 0.38 for **PE-S**. Interestingly, increasing the oxidation state further to the corresponding sulfones in **PE-SO2** leads to a *Φ* value of 0.37. The evolution in *Φ* values suggests that as the sulfide is oxidized to sulfoxide and sulfone, the relative mechanical strength of C–S bonds in each polymer follows the order: **PE-S** ∼ **PE-SO2** > **PE-SO**. A rough way to evaluate this outcome is comparing the C–S bond dissociation energy (BDE), for the reason that mechanically induced bond cleavage is essentially a force-assisted bond dissociation. Prior work by others suggest that the C–S BDEs of sulfide, sulfoxide, and sulfone are 74–77 kcal mol^−1^, 53–54 kcal mol^−1^, and ∼68 kcal mol^−1^, respectively.^33^ These BDE values are substantially smaller than those of conventional C–C (>80 kcal mol^−1^) or C–O (>90 kcal mol^−1^)^48,49^ bonds, and indicate that the chain scission preferentially occurs at the C–S bond along the polymer backbone. That the sulfur species are responsible for chain scission is supported by two pieces of evidence. First, a previous study of the polyester obtained from copolymerization of *g*DCC and glutaric acid showed that *Φ* = 0.63 for that polymer, even though the polymer in question had a much higher molecular weight (*M*_n_ = 140 kDa) and longer contour length than the polymer employed here (higher contour length corresponds to lower *Φ*).^37^ Second, CoGEF simulations of extension lead to scission of the C–S bond in all species (see ESI†).

The relative mechanical strength (**PE-S** ∼ **PE-SO2** > **PE-SO**) is aligned with the BDEs of the various C–S bonds. We computed the relative BDEs of the C–S bonds within the sulfide, sulfoxide and sulfone. The calculation was performed using DFT method on theory level of B3LYP/6-311+G** (details provided in ESI†). As shown in Table 1, the calculated BDEs of C–S bonds follows the order of sulfide > sulfone > sulfoxide, and the relative values agree both with prior work^33^ and with the relative mechanical strengths inferred from the ultrasonication study. Robb and co-workers^50^ have recently reported good agreement between the peak force sustained by CoGEF calculations and the propensity of a given bond to break, and a similar trend is observed here (see Table S1, ESI†). In addition, a very recent study by Diesendruck *et al.*^51^ on the impact of intramolecular crosslinker on the mechanochemical fragmentation of covalently folded polymers found that the sulfone crosslinkers are slightly more prone to mechanical fragmentation than are sulfide crosslinkers. The results here are consistent with the observations by Diesendruck and co-workers reflecting the intrinsic mechanical strength of the crosslinkers rather than differences in the shape of collapsed chains brought about by the polarity of the sulfone.

**Table 1:** Computational bond dissociation energies (BDE) of C–S bonds in sulfide, sulfoxide and sulfone. Calculations were set in continuum dielectric of 7.43 (simulating a nonpolar solvent)

C–S bond	Sulfide	Sulfoxide	Sulfone
BDE (kcal mol^−1^)	68	46	60

## Conclusions

In conclusion, the above results demonstrate a straightforward approach by which to regulate the mechanical strength of C–S bonds in polymers through oxidation reactions. Combined with the wide range of strategies to embed sulfides in polymers, this result facilitates the preparation of polymeric materials in which the mechanical response of C–S bonds to an external oxidant alters the strength and toughness of a single polymer chain. We reason that the ability to attain *in situ* switching of mechanochemical scission provides a means to test models that connect single molecular and bulk properties. Looking ahead, a promising opportunity for this and similar methodologies lies in testing molecular theories of polymer fracture behavior (*e.g.*, Lake–Thomas theory^2^), in which the energy dissipation can be correlated to single chain toughness.^1^

## Conflicts of interest

There are no conflicts to declare.

## Supplementary Material

SC-011-D0SC04381H-s001
