# Supplementary material for: Oxidative regulation of the mechanical strength of a C–S bond
Source: Chem Sci. 2020 Sep 14;11(38):10444–8. doi: 10.1039/d0sc04381h (PMC8162406; doi:10.1039/d0sc04381h)
Supplement: SC-011-D0SC04381H-s001 [file SC-011-D0SC04381H-s001.pdf]

Electronic Supplementary Material (ESI) for Chemical Science.
This journal is © The Royal Society of Chemistry 2020

Supporting Information

Oxidative regulation of the mechanical strength of a C-S bond

Yangju Lin and Stephen L. Craig*

Department of Chemistry, Duke University, Durham, North Carolina 27708, United States

Contents

I. General procedures 2
Materials 2
Characterizations 2
II. Synthesis procedures 2
1. Synthesis of polymer PE-S 3
2. Synthesis of polymer PE-SO 3
3. Synthesis of polymer PE-SO_2 3
III. Sonication experiment 3
1. General sonication procedures 3
2. Sonication of PE-S polymer 4
1) Calculation of **PE-S** polymer chain scission cycle 4
2) Determination of ring-opening percentage of *g*DCC in **PE-S** polymer 4
3) Evaluation of Φ value 5
3. Sonication of PE-SO polymer 6
1) Calculation of **PE-SO** polymer chain scission cycle 6
2) Determination of ring-opening percentage of *g*DCC in **PE-SO** polymer 7
3) Evaluation of Φ value 7
4. Sonication of PE-SO_2 polymer 8
1) Calculation of **PE-SO_2** polymer chain scission cycle 8
2) Determination of ring-opening percentage of *g*DCC in **PE-SO_2** polymer 9
3) Evaluation of Φ value 10
IV. CoGEF modeling 10
1. CoGEF modeling of sulfide repeating unit 10
2. CoGEF modeling of sulfoxide repeating unit 10
3. CoGEF modeling of sulfone repeating unit 11
V. Calculation of bond dissociation energy 12
VI. ^{1}H NMR spectrum 13
VII. Reference 14

I. General procedures

Materials

Lab general solvents (dichloromethane, ethyl acetate, acetone, toluene, tetrahydrofuran, methanol) were purchased from VWR or Sigma Aldrich. 4-Dimethylaminopyridine, *p*-toluenesulfonic acid, N,N'-diisopropylcarbodiimide, 3,3'-thiodipropionic acid, glutaric acid, hydrogen peroxide, acetic anhydride, *meta*-chloroperoxybenzoic acid (mCPBA) and silica dioxide were purchased from Sigma Aldrich, Alfa Aesar or TCI and used without further purification.

Characterizations

^{1}H NMR spectra were collected on a Bruker Advance Neo - 500 MHz multinuclear NMR spectrometer. Chemical shifts are provided in *ppm* (δ) and referenced to the residual ^{1}H peak at 7.26 ppm in $CDCl_3$. ^{1}H shifts are reported as chemical shift, multiplicity, coupling constant if applicable, and relative integral. Multiplicities are reported as: singlet (s), doublet (d), doublet of doublets (dd), doublet of triplets (dt), doublet of doublet of doublets (ddd), doublet of doublet of triplets (ddt), triplet (t), triplet of doublets (td), quartet (q), pentet (p), multiplet (m), or broad (br). Coupling constants (J) are reported in Hz. Gel permeation chromatography (GPC) was performed on two Agilent PLgel mixed-C columns (10^5 Å, 7.5x300 mm, 5 µm, part number PL1110-6500) using THF (stabilized with 100 ppm BHT) as the eluent. Molecular weights were calculated using a Wyatt Dawn EOS multi-angle light scattering (MALS) detector and Wyatt Optilab DSP Interferometric Refractometer (RI). The refractive index increment (dn/dc) values were determined by online calculation based on injections of known concentration and mass.

II. Synthesis procedures

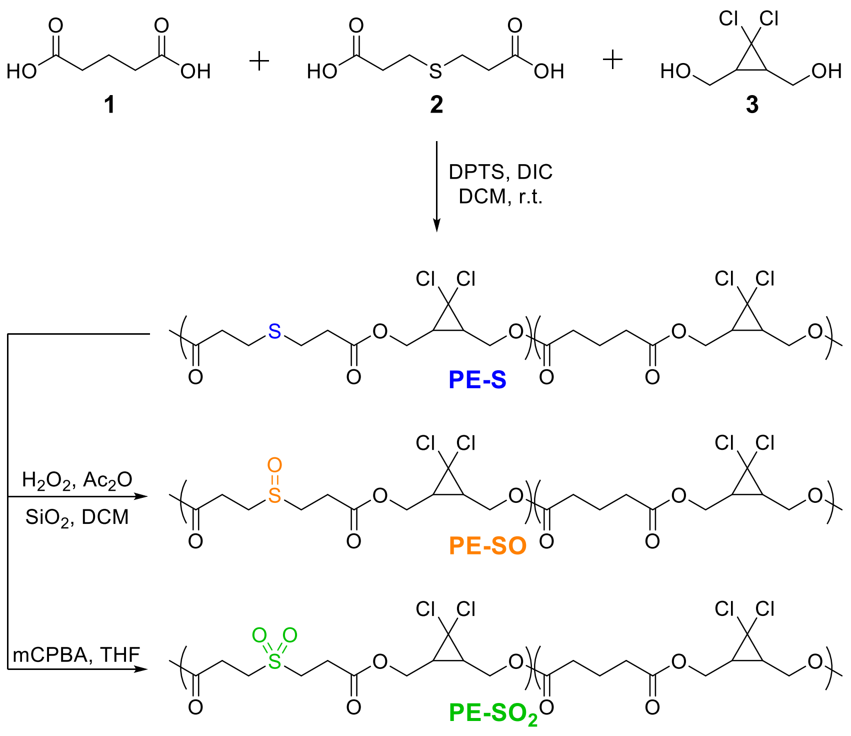

1. Synthesis of polymer PE-S

To a 50 mL oven dried round bottom flask, added glutaric acid **1** (528.4 mg, 4.0 mmol, 0.8 eq), 3,3'-thiodipropionic acid **2** (178.2 mg, 1.0 mmol, 0.2 eq), *gem*-dichlorocycloprpane (gDCC) diol **3**[1] (855 mg, 5.0 mmol, 1.0 eq) and 4-(dimethylamino)pyridinium-4-toluenesulfonate (DPTS)[2] (588 mg, 2.0 mmol, 0.4 eq) as catalyst. 5 mL dry DCM was then added to the flask. After solids are completely dissolved, N, N'-diisopropylcarbodiimide (DIC) (2.32 mL, 15 mmol, 3.0 eq) was added dropwise to the solution. White precipitate formed during the addition. The flask was then sealed and allowed to stir at room temperature for 3 days. The resulting viscous mixture was precipitated from methanol three times. Obtained white gummy polymer **PE-S** was collected and dried under high vacuum (1.132 g, 82%). Obtained polymer was analyzed from GPC: M_n= 71.7 kDa, $Đ_M$ = 1.445, dn/dc= 0.087. ^{1}H NMR (500 MHz, $CDCl_3$) δ: 4.33-4.15 (m, 10H), 2.84 (t, 2H), 2.68 (t, 2H), 2.45 (t, 8H), 2.20-2.06 (m, 5H), 1.99 (p, 4H). Calculated sulfide content: 2/10×100% = 20 mol%.

Another **PE-S** polymer with M_n = 60.0 kDa ($Đ_M$ = 1.437) was prepared using the same procedure.

2. Synthesis of polymer PE-SO

The oxidation process was adapted from previous literature.[3]

Polymer **PE-S** (78 mg, M_n = 71.7 kDa, 0.05 mmol C-S bond) was weighted in a 10 ml scintillation vial. 1 mL DCM was added to completely dissolve the polymer. Acetic anhydride (5.2 µL, 0.055 mmol, 1.1 eq) and silica gel (50 mg) was added to the solution. Then, 30% hydrogen peroxide (57 µL, 0.5 mmol, 10 eq) was added to the mixture. The reaction was further stirred at room temperature for 6 h. The resulting mixture was then diluted with 2 mL DCM and silica gel was filtered using a syringe filter. Obtained DCM solution was condensed and precipitated from methanol three time to give polymer **PE-SO** (74 mg, 93.9%). GPC: M_n = 59.9 kDa, $Đ_M$ = 1.478, dn/dc = 0.090. ^{1}H NMR (500 MHz, $CDCl_3$) δ: 4.36-4.16 (m, 10H), 3.16-3.05 (m, 1H), 3.01-2.93 (m, 1H), 2.93-2.84 (m, 2H), 2.45 (t, 8H), 2.20-2.06 (m, 5H), 1.99 (p, 4H). Calculated sulfoxide content: 1.9/10×100% = 19 mol%.

3. Synthesis of polymer PE-SO_2

To a solution of **PE-SO** (156 mg, M_n = 71.7 kDa, 0.1 mmol C-S bond) in 3 mL THF, mCPBA (74 mg, 70%~75%, 0.3 mmol) was added in portions. The reaction was allowed to stir at room temperature overnight. After the reaction completed, the solution was condensed and precipitated from methanol three times to give a white polymer **PE-SO_2** (158 mg, 99.9%). GPC: M_n = 54.1 kDa, $Đ_M$ = 1.478, dn/dc = 0.091. ^{1}H NMR (500 MHz, $CDCl_3$) δ: 4.37-4.16 (m, 10H), 3.40 (t, 2H), 2.94 (t, 2H), 2.45 (t, 8H), 2.20-2.06 (m, 5H), 1.99 (p, 4H). Calculated sulfone content: 2/10×100% = 20 mol%.

III. Sonication experiment

1. General sonication procedures

A solution of 36 mg polymer (**PE-S**, **PE-SO** or **PE-SO_2**) in 18 mL dry THF (c = 2 mg/mL) was transferred into a Suslick cell. The solution was purged with N_2 for 10 min while cooled with ice bath. Pulsed ultrasound was applied (1s on, 1s off) at 30% amplitude. An aliquot of 0.8 mL sample was taken out for GPC analysis at each sonication time points: 0, 3, 6, 10, 15, 20, 30, 45 min. Each of these samples were further condensed in a 10 ml scintillation vial to give a thin layer of polymer at the vial bottom. The polymer was washed with methanol and further dried under high vacuum. Obtained dry polymers were further subjected to ^{1}H NMR analysis.

2. Sonication of PE-S polymer

1) Calculation of **PE-S** polymer chain scission cycle

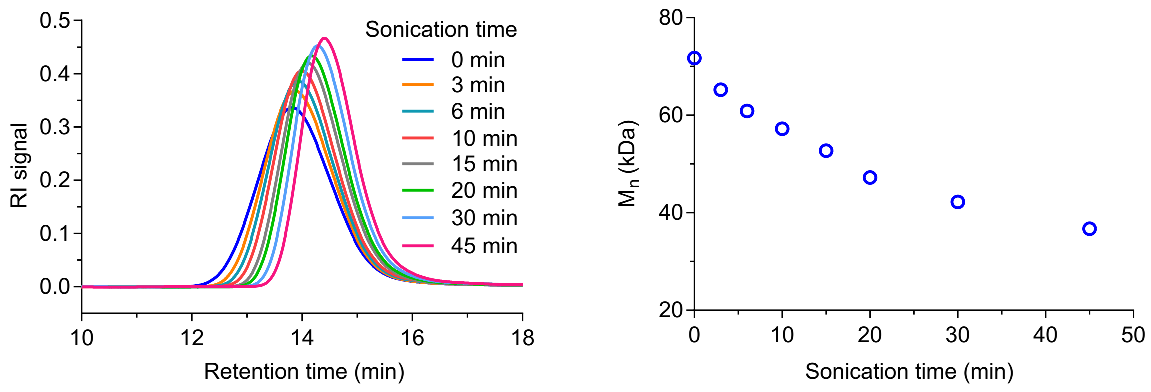

Figure S1. Evolution of GPC traces during sonication of **PE-S** polymer (left) and corresponding plot of molecular weight versus sonication time (right).

The chain scission cycle (**SC**) describes the average number of times a polymer has undergone chain scission. **SC** of **PE-S** polymer at each sonication time was obtained from the following equation:

$$\mathbf{SC} = \frac{\ln M_{n(0)} - \ln M_{n(t)}}{\ln 2}$$

Where $M_{n(o)}$ is the initial molecular weight; $M_{n(t)}$ is sonicated molecular weight.

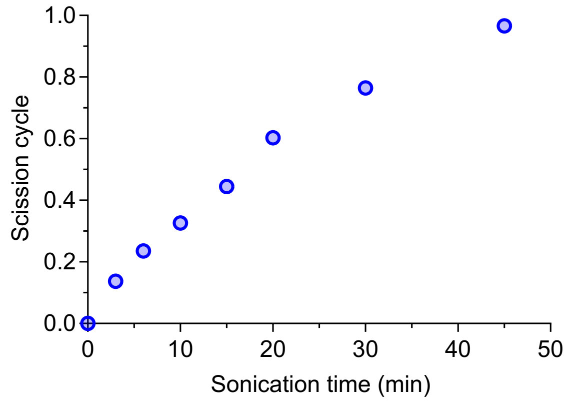

Figure S2. Scission cycle (**SC**) of **PE-S** polymer at various sonication times.

2) Determination of ring-opening percentage of *g*DCC in **PE-S** polymer

In the ^{1}H NMR spectrum, the peak at 4.17–4.32 ppm (**H$_e$**) corresponds to mehthlene protons adjacent to the cyclopropane in the pristine polymer. The peak at 6.13 ppm (**H$_a$**), 4.68–4.85 ppm (**H$_b$&H$_c$**), and 6.39 ppm (**H$_d$**) are protons in the ring opened *g*DCC product. The ring-opening percentage can be obtained:

$$\text{Ring opening } \% = \frac{4 * \int H_a}{\int H_b + \int H_d + \int H_e} \times 100\%$$

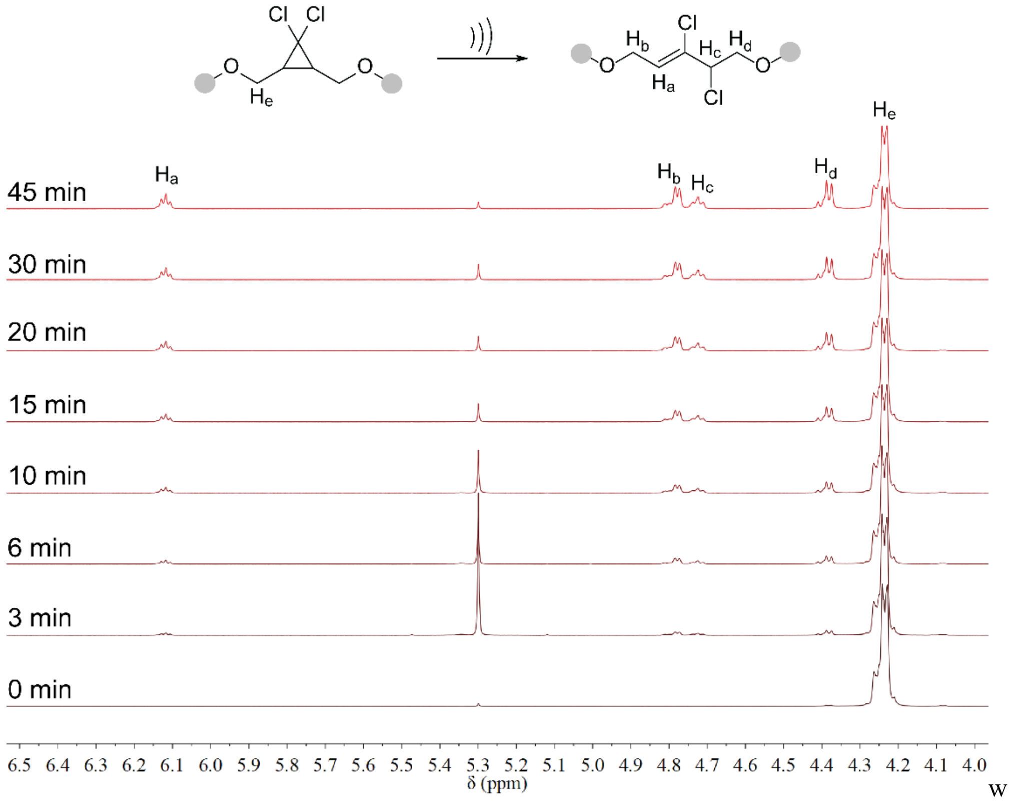

Figure S3. Stack of ^{1}H-NMR ($CDCl_3$, 500 MHz) spectra of sonicated **PE-S** polymer at various sonication times.

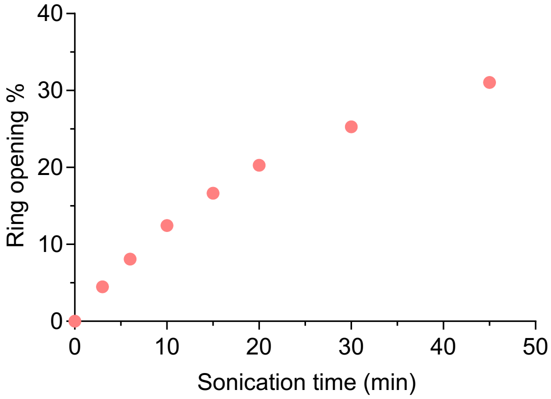

Figure S4. Ring-opening percentage of *g*DCC in **PE-S** polymer versus sonication time.

3) Evaluation of Φ value

Φ value, the slope of ring opening percentage of *g*DCC versus scission cycle (**SC**) plot, is used to evaluate the relative strength of C-S weak bonds.

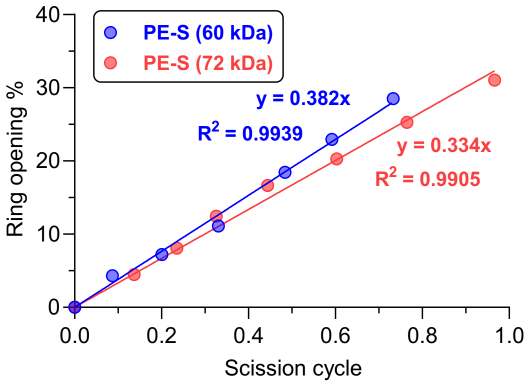

Figure S5. Percentage of *g*DCC ring opening versus scission cycle plot for **PE-S** polymer. The slope of linear fitting through origin gives Φ = 0.334 for M_n = 72 kDa and Φ = 0.382 for M_n = 60 kDa.

3. Sonication of PE-SO polymer

The analysis of **PE-SO** polymer is the same as **PE-S** polymer.

1) Calculation of **PE-SO** polymer chain scission cycle

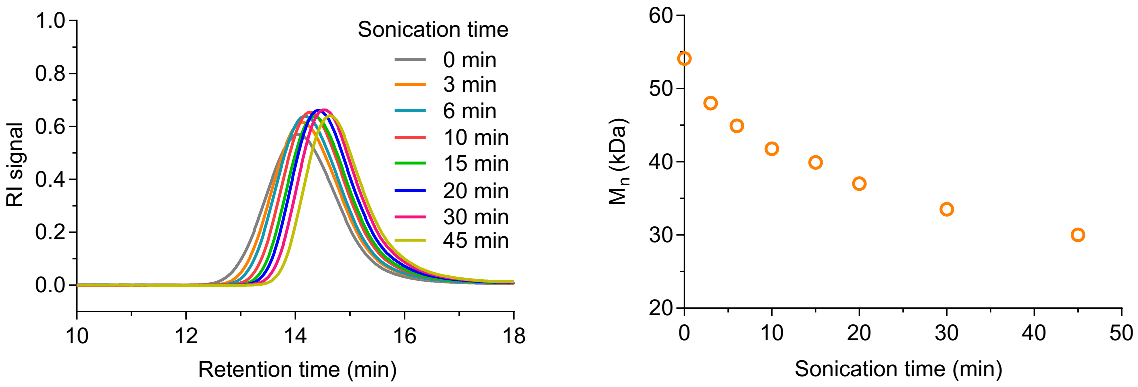

Figure S6. Evolution of GPC traces during sonication of **PE-SO** polymer (left) and corresponding plot of molecular weight versus sonication (right).

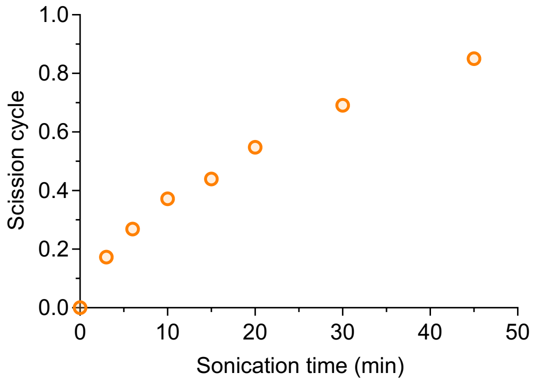

Figure S7. Scission cycle (**SC**) of **PE-SO** polymer at various sonication time

2) Determination of ring-opening percentage of *g*DCC in **PE-SO** polymer

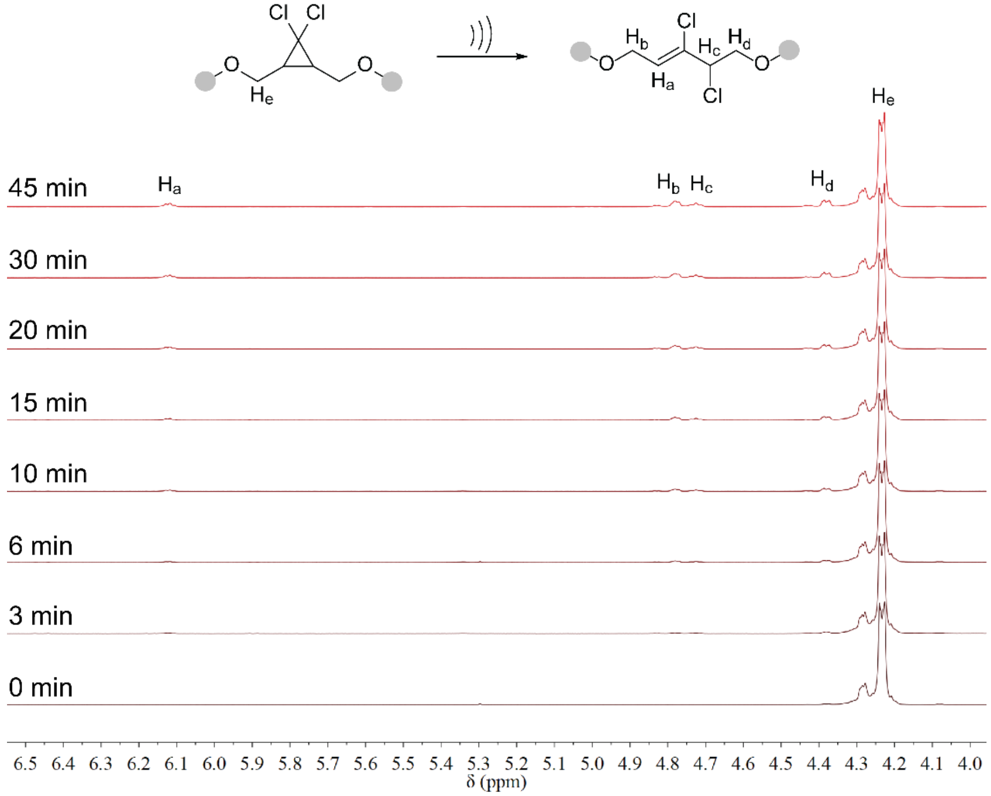

Figure S8. Stack of ^{1}H-NMR ($CDCl_3$, 500 MHz) spectra of sonicated **PE-SO** polymer at various sonication times.

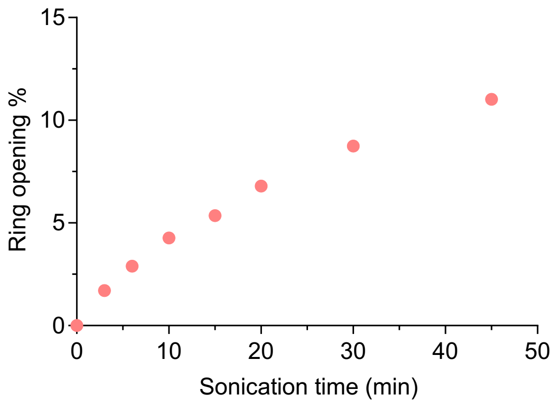

Figure S9. Ring-opening percentage of *g*DCC in **PE-SO** polymer versus sonication time.

3) Evaluation of Φ value

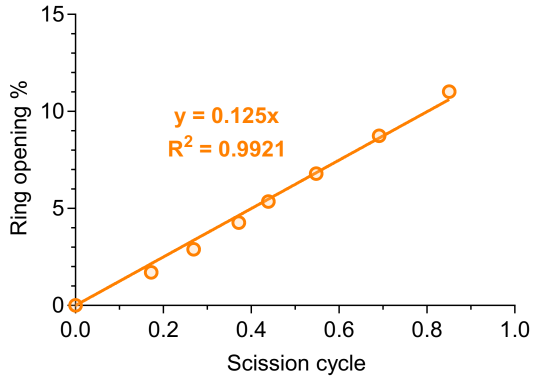

Figure S10. Percentage of *g*DCC ring opening versus scission cycle plot for **PE-SO** polymer. The slope of linear fitting through origin gives Φ = 0.125.

4. Sonication of PE-SO_2 polymer

The procedure of analyzing **PE-SO_2** polymer is the same as **PE-S** polymer.

1) Calculation of **PE-SO_2** polymer chain scission cycle

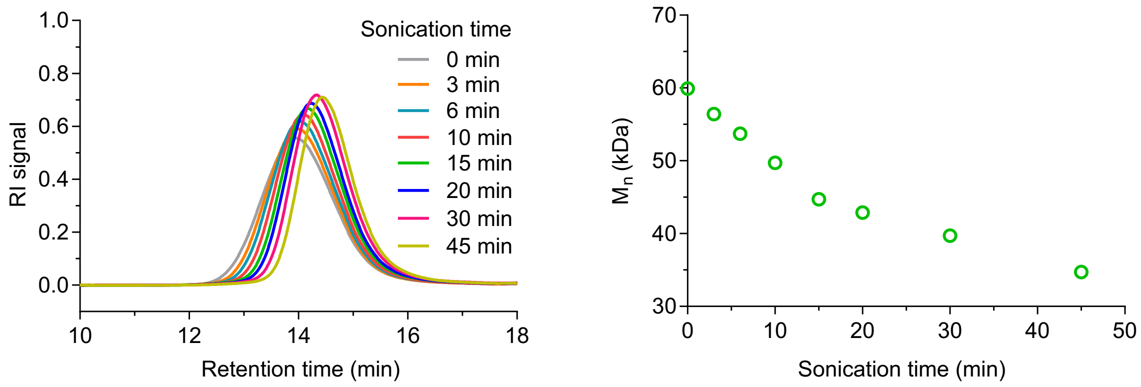

Figure S11. Evolution of GPC traces during sonication of **PE-SO** polymer (left) and corresponding plot of molecular weight versus sonication (right).

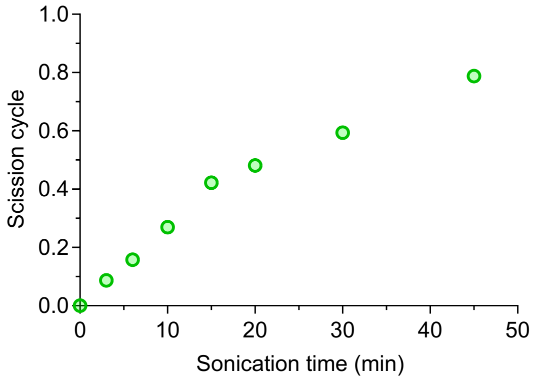

Figure S12. Scission cycle (**SC**) of **PE-SO** polymer at various sonication time

2) Determination of ring-opening percentage of *g*DCC in **PE-SO₂** polymer

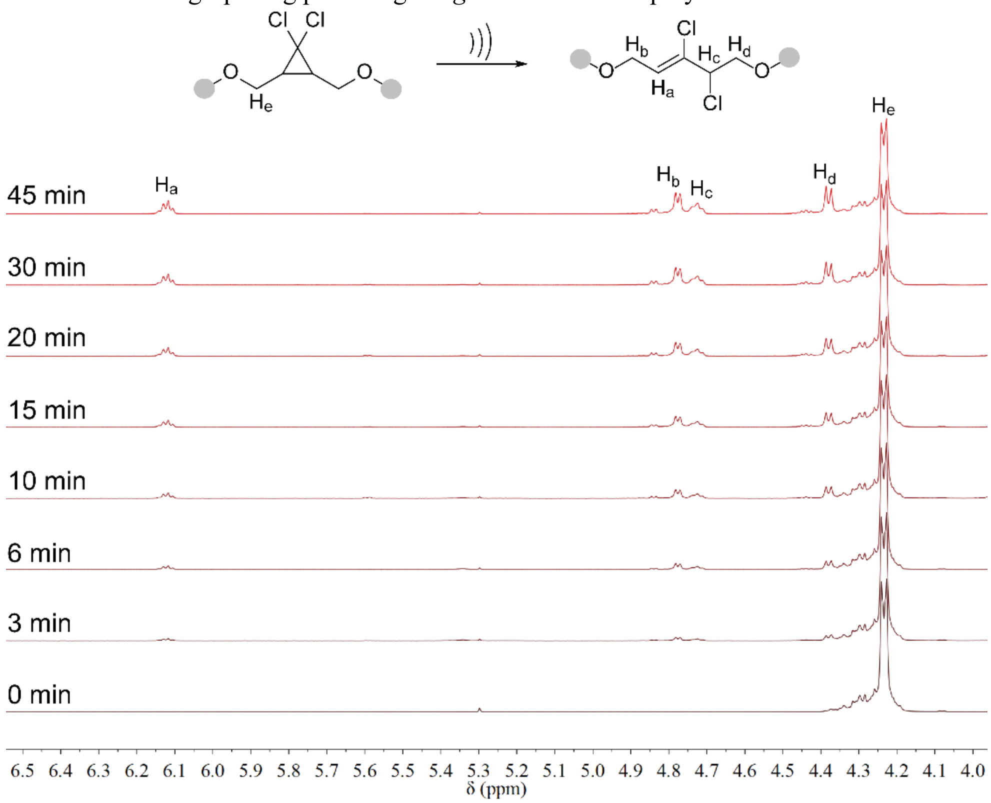

Figure S13. Stack of ^{1}H-NMR ($CDCl_3$, 500 MHz) spectra of sonicated **PE-SO₂** polymer at various sonication times.

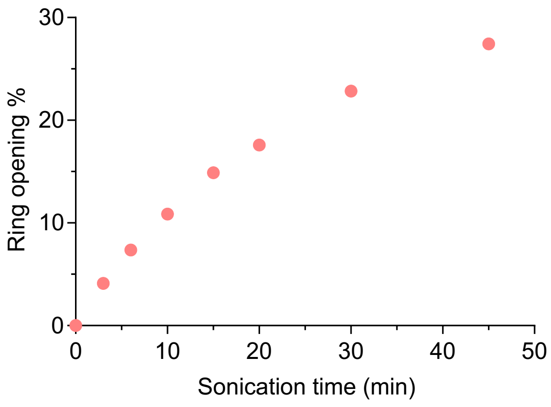

Figure S14. Ring-opening percentage of *g*DCC in **PE-SO₂** polymer versus sonication time.

3) Evaluation of Φ value

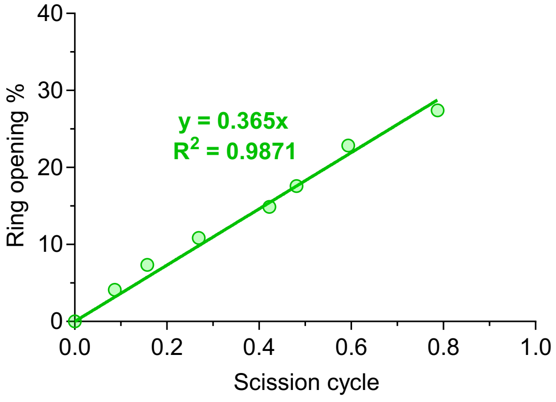

Figure S15. Percentage of *g*DCC ring opening versus scission cycle plot for **PE-SO₂** polymer. The slope of linear fitting through origin gives Φ = 0.365.

IV. CoGEF modeling

CoGEF modeling was conducted using Spartan '18 V1.4.1 software. The methyl ester form of each sulfide, sulfoxide or sulfone containing repeating unit was subjected to CoGEF modeling using DFT method on the theory level of B3YLP/61G*. The ground state geometry was first optimized, and then its end-to-end distance was constrained with step increasement of 0.1 Å. The optimized energy was plotted as a function of end-to-end distance.

1. CoGEF modeling of sulfide repeating unit

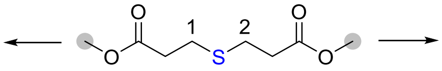

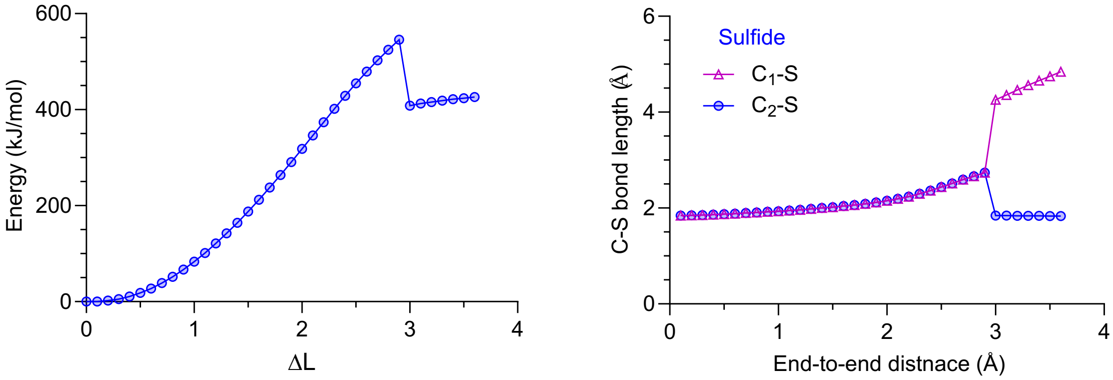

Figure S16. CoGEF modeling of sulfide containing unit. Left: the evolution of energy over increasement in end-to-end distance. Right: the length of two C-S bonds at different constrained distance.

2. CoGEF modeling of sulfoxide repeating unit

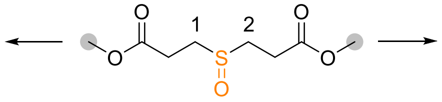

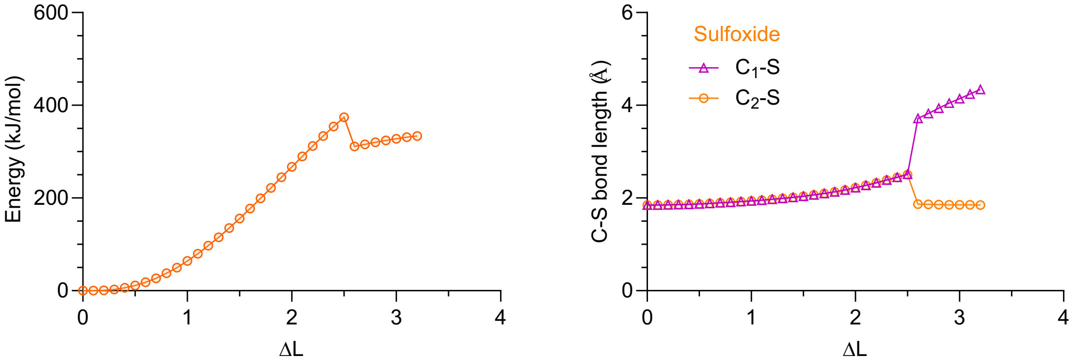

Figure S17. CoGEF modeling of sulfoxide containing unit. Left: the evolution of energy over increasement in end-to-end distance. Right: the length of two C-S bonds at different constrained distance.

3. CoGEF modeling of sulfone repeating unit

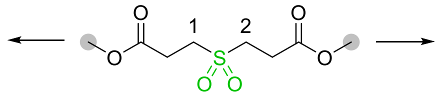

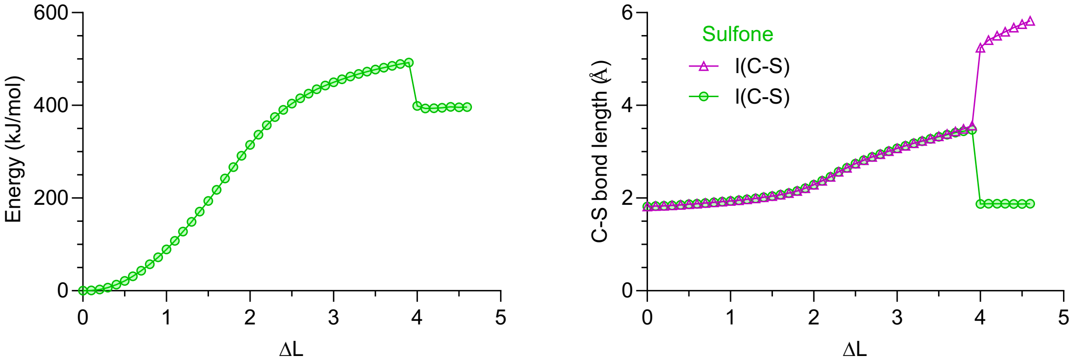

Figure S18. CoGEF modeling of sulfone containing unit. Left: the evolution of energy over increasement in end-to-end distance. Right: the length of two C-S bonds at different constrained distance.

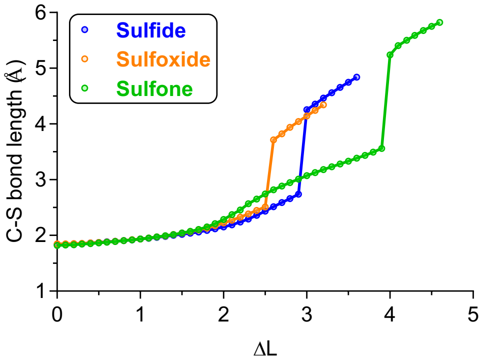

Figure S19. Overlay of length of breaking C-S bonds sdfs at various constrained distance.

Table S1. Summary of F_{max} obtained from GoGEF modeling of C-S bond containing species

C-S bond	Sulfide	Sulfoxide	Sulfone
F_{max} (nN)	4.61	3.78	4.07

V. Calculation of bond dissociation energy

The methyl ester form of each sulfide, sulfoxide or sulfone containing repeating unit was applied to calculate the C-S bond dissociation energy (BDE). The BDE was calculated using equation: BDE = E(Frag1) + E(Frag2) - E(SM).

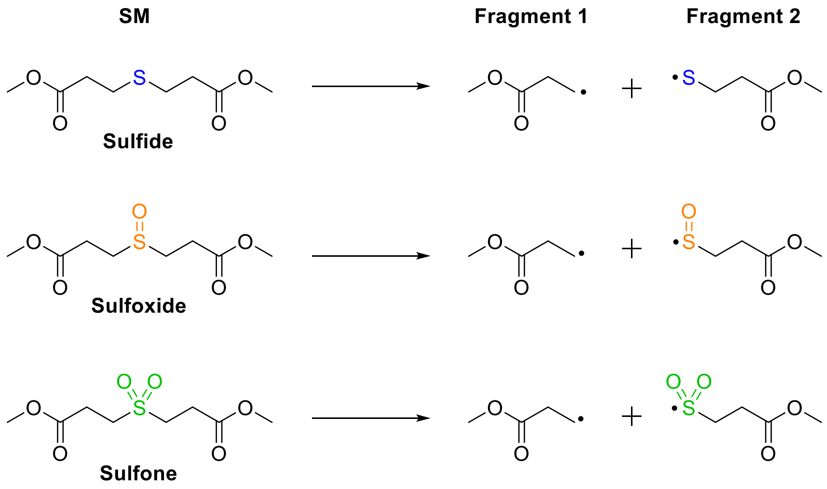

The calculation was performed in Spartan '18 V1.4.1 software. The energy of each starting molecule was optimized using DFT method on theory level of B3LYP/6-311+G** (in nonpolar solvent, $\varepsilon = 7.43$), and the two radical fragments were optimized using the same method but with one unpaired electron. The obtained optimized energy for each molecule/fragment was provided in the following table.

Table S2. Summary of calculated bond dissociation energy (BDE) for C-S bond containing species

	Optimal energy (E_h)			BDE	
	SM	Fragment 1	Fragment 2	E_h	kcal/mol
**Sulfide**	-1012.61727	-307.133215	-705.375901	0.10815	67.9
**Sulfoxide**	-1087.82834	-307.133215	-780.621622	0.07350	46.1
**Sulfone**	-1163.06402	-307.133215	-855.834720	0.09609	60.3

VI. ^{1}H NMR spectrum

PE-S

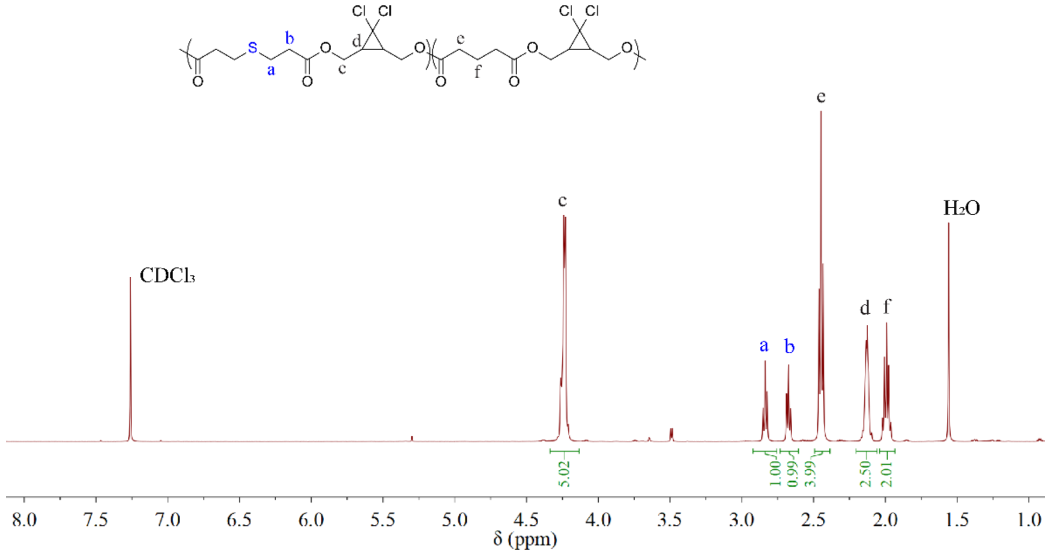

^{1}H NMR ($CDCl_3$, 500 MHz) spectrum of **PE-S** polymer

PE-SO

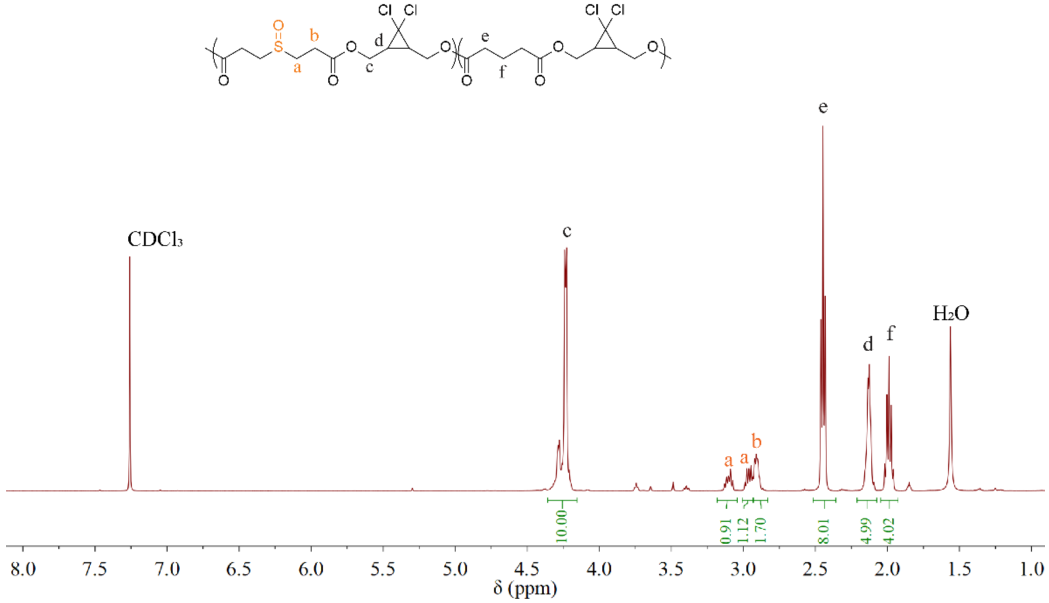

^{1}H NMR ($CDCl_3$, 500 MHz) spectrum of **PE-SO** polymer

PE-SO_2

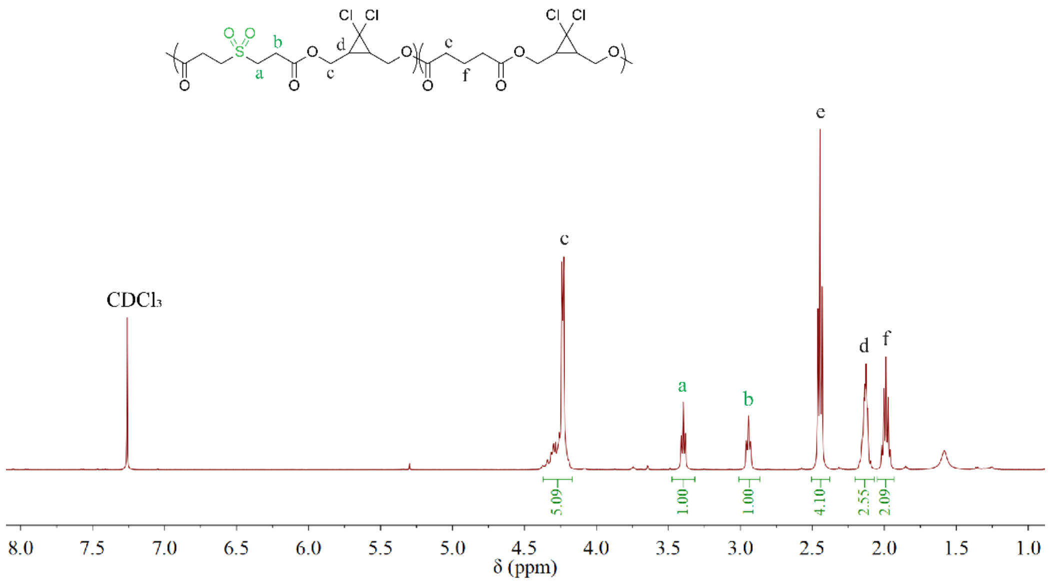

^{1}H NMR ($CDCl_3$, 500 MHz) spectrum of **PE-S** polymer

VII. Reference

1. Kean, Z. S.; Black Ramirez, A. L.; Craig, S. L., High Mechanophore Content Polyester-Acrylate ABA Block Copolymers: Synthesis and Sonochemical Activation. *J Polym Sci B Polym Phys* **2012,** *50* (17), 3481-3484.
2. Moore, J. S.; Stupp, S. I., Room-Temperature Polyesterification. *Macromolecules* **1990,** *23* (1), 65-70.
3. Kakarla, R.; Dulina, R. G.; Hatzenbuhler, N. T.; Hui, Y. W.; Sofia, M. J., Simple and Efficient Method for the Oxidation of Sulfides to Sulfoxides: Application to the Preparation of Glycosyl Sulfoxides. *J Org Chem* **1996,** *61* (23), 8347-8349.
4. Lee, B.; Niu, Z.; Wang, J.; Slebodnick, C.; Craig, S. L., Relative Mechanical Strengths of Weak Bonds in Sonochemical Polymer Mechanochemistry. *J Am Chem Soc* **2015,** *137* (33), 10826-32.
5. Lenhardt, J. M.; Black Ramirez, A. L.; Lee, B.; Kouznetsova, T. B.; Craig, S. L., Mechanistic Insights into the Sonochemical Activation of Multimechanophore Cyclopropanated Polybutadiene Polymers. *Macromolecules* **2015,** *48* (18), 6396-6403.
